# Adipocytes from the infrapatellar fat pad of OA patients modulate CD4+ T cell cytokine production

**DOI:** 10.1186/1479-5876-9-S2-P45

**Published:** 2011-11-23

**Authors:** Andreea Ioan-Facsinay, Joanneke C Kwekkeboom, Margreet Kloppenburg, Rene EM Toes

**Affiliations:** 1 Dept. of Rheumatology, Leiden University Medical Center, Leiden, The Netherlands

## Aim

As shown in previous studies, the infrapatellar fat pad (IFP) of OA patients has an inflammatory phenotype and is infiltrated with immune cells, including CD4+ T cells. Because several studies have shown that the phenotype of CD4+ T cells in adipose tissue is changing with the adiposity of the individual, we hypothesized that this effect could be due to the modulation of infiltrating T cells by the tissue-resident adipocytes. Therefore, we investigated whether adipocytes from IFP can modulate CD4+ T cell cytokine production.

## Methods

CD4+ T cells were purified from peripheral blood mononuclear cells isolated from healthy volunteers. The purity of the CD4+ T cells isolated using magnetic beads coated with anti-human CD4 was above 95%. Plate-bound anti-CD3 and soluble anti-CD28 antibodies were used to activate T cells. Adipocytes were isolated from IFP of OA patients by collagenase digestion and were either cultured with purified CD4+ T cells or were cultured in vitro for 24 hours in DMEM/F12 medium supplemented with 0.5% bovine serum albumin to generate adipocyte-conditioned medium (ACM). Cytokine production was measured by intracellular cytokine staining (ICS), ELISA or cytokine multiplex. Separation of the protein and lipid fractions was performed using the Bligh and Dyer method, followed by precipitation of the protein fraction and reconstitution in culture medium.

## Results

CD4+ T cells produced increase levels of IFNγ when activated in the presence of adipocytes. This effect is mediated by soluble mediators, as shown in transwell and ACM transfer experiments. Moreover, separation of the protein and lipid fractions from the ACM medium showed that soluble factors present in the protein fraction can mediate this effect. Measurement of ten different cytokines known to be secreted by T cells upon activation revealed that, besides IFNγ, also TNFα, IL-17 and IL-5 are modulated by IFP-derived ACM. Finally, CD4+ T cells from IFP produced more IFNγ than blood CD4+ T cells from paired samples.

## Conclusions

Soluble mediators secreted by IFP of OA patients can modulate cytokine production by CD4+ T cells. These mediators are contained in the protein fraction in at least some patients.

